# Intraventricular intracranial pressure monitoring improves the outcome of older adults with severe traumatic brain injury: an observational, prospective study

**DOI:** 10.1186/s12871-016-0199-9

**Published:** 2016-07-11

**Authors:** Wendong You, Junfeng Feng, Qilin Tang, Jun Cao, Lei Wang, Jin Lei, Qing Mao, Guoyi Gao, Jiyao Jiang

**Affiliations:** 1 Department of Neurosurgery, Renji Hospital, School of Medicine, Shanghai Jiao Tong University, Shanghai, 200127 People’s Republic of China; 2 Shanghai Institute of Head Trauma, Shanghai, 200127 People’s Republic of China

**Keywords:** Intraventricular intracranial pressure monitoring, Severe traumatic brain injury, Older adults

## Abstract

**Background:**

Intracranial pressure (ICP) monitoring is widely used in the management of patients with severe traumatic brain injury (TBI). However, there is limited evidence about the efficacy of ICP monitoring in older subjects (aged ≥65 years). This study evaluated the effect of intraventricular ICP monitoring on the outcome of older adults suffering from a severe TBI.

**Methods:**

This prospective, observational study included 166 older TBI patients (aged ≥65 years) with Glasgow Coma scale (GCS) scores lower than 9 at admission. The study cohort was divided into two groups, intraventricular ICP monitoring and non-ICP monitoring. The primary outcome was in-hospital mortality. The secondary outcomes included the Glasgow Outcome Scale (GOS) score 6 months after injury, the ICU and total hospital lengths of stay, and mechanical ventilation days.

**Results:**

There were 80 patients in the intraventricular ICP monitoring group and 86 patients in non-ICP monitoring group. There was no statistical difference between groups in demographics and severity of head injury. Patients treated with intraventricular ICP monitoring had lower in-hospital mortality (33.8 % vs 51.2 %, *P* < 0.05), a higher 6-month GOS score (3.0 ± 1.4 vs 2.5 ± 1.2 *P* < 0.05), and a lower dosage (514 ± 246 g vs 840 ± 323 g, *P* < 0.0001) and shorter duration (7.2 ± 3.6 days vs 8.4 ± 4.3 days, *P* < 0.01) of mannitol use. However, the ICU length of stay (14.3 ± 6.4 days vs 11.6 ± 5.8 days, *P* < 0.01) and mechanical ventilation days (6.7 ± 3.5 days vs 5.6 ± 2.4 days, *P* < 0.05) were longer in the ICP monitoring group. The total length of hospital stay did not differ between the two groups (28.5 ± 12.1 days vs 26.1 ± 13.5 days, *P* = 0.23).

**Conclusions:**

Intraventricular ICP monitoring may have beneficial effects on the decreased in-hospital mortality and improved 6-month outcome of older patients with severe TBI. However, given that this was an observational study conducted in a single institution, further well-designed randomized control trials are needed to evaluate the effect of intraventricular ICP monitoring on the outcome of older severe TBI patients.

## Background

Traumatic brain injury (TBI) is a social and economic problem worldwide. It is the leading cause of death and disability among young individuals [1]. However, with the aging population, the proportion of older people (aged ≥65 years) with a TBI is expected to increase significantly in the United States and other developed countries [2]. Evidence suggests that in the United States TBI-related hospital visits among older adults are increasing at a rate that exceeds the population growth [3]. It is widely acknowledged that older adults who suffered from a TBI tend to have poorer outcome and slower recovery than younger ones even after milder injuries [2, 4]. The poorer outcomes observed in older patients suggest that the clinical condition of TBI in older adults may be different from that of the younger population; thus, the older group of patients may require special treatment for a TBI. However, age-specific treatment guidelines for TBI do not currently exist.

Intracranial pressure (ICP) monitoring is considered the standard of care for severe TBI and has been recommended by several guidelines [5–7]. Although, the efficacy of ICP monitoring-based treatment remains controversial, some studies showed improved outcome [8–10] with ICP monitoring; while, others reported no benefits [11, 12] or even worsening outcomes [13]. Recent accumulating evidence suggested ICP monitoring only benefits some subgroups or subtypes of TBI patients. Therefore, it is necessary to identify these subgroups [14, 15]. In older adults, TBI differs in multiple ways from other age groups, even those experiencing the same injuries [3]. To the best of our knowledge, few studies have examined the role of ICP monitoring in this subgroup of TBI patients. Intraventricular ICP monitoring is widely used in clinical practice because it is accurate and reliable. It also provides an option of lowering ICP by draining cerebrospinal fluid (CSF) [16, 17]. The aim of this study was to evaluate the effect of intraventricular ICP monitoring on the outcome of older individuals with severe a TBI.

## Methods

### Protocol approval and patient consent

This observational, prospective study was conducted in the neurosurgical department of Renji Hospital, affiliated with Shanghai Jiao Tong University School of Medicine. The protocol of the present study was approved by the Ethics Committee of Shanghai Renji Hospital. Verbal informed consent was obtained for all follow-up interviews by phone, and written informed consent was obtained for outcome assessments via postal questionnaire.

### Sample size calculation

Sample size was calculated using two proportions power analysis on the basis of the primary outcome measure. It was estimated that 80 patients per group would be required for the power analysis. Power (1-β) was set at 0.80, and the type I error (α) was set at 0.1.

### Patients

All patients presenting with a TBI from January 2008 to June 2014 in the study hospital were screened for eligibility. Inclusion criteria were a) aged ≥65 years; b) history of acute TBI; c) Glasgow Coma Scale (GCS) score lower than 9 at admission; and d) initial computed tomography (CT) scan showed intracranial abnormalities consistent with head trauma. Patients who died within 24 h of brain injury or were admitted with a diagnosis of brain death were excluded from the study. Those admitted to our department 24 h after sustaining the injury were also excluded.

### Study group and treatment protocol

Subjects in this study were arranged in two groups, the ICP monitoring group (using an intraventricular ICP monitor) and non-ICP monitoring group (control group). Indications for ICP monitoring were: a) severe TBI with an abnormal CT scan at presentation or b) severe TBI with a normal CT scan and the presence of two or more of the following features at admission: age older than 40 years, motor posturing, or systolic blood pressure lower than 90 mmHg [18]. Although guidelines on ICP monitoring have been adopted by our department, its implementation was not universal. Patients in the control group met the criteria of ICP monitoring, but were not monitored for several reasons, including: the judgment and experience of the neurosurgeons, the patient’s or their caregiver’s decision to receive more conservative treatment, and the limited availability of monitoring devices and trained staff for inserting the ICP monitor.

Patients with a TBI were managed according to a standardized protocol based on the guidelines set up by the Brain Trauma Foundation. For patients in both groups, the clinical neurological status (GCS score, pupil size, and reactivity) was monitored hourly. Head CT were obtained at admission, 48 h, 5 to 7 days after admission, and any time as needed based on the clinical condition. Invasive mean arterial pressure was measured and maintained between 70 mmHg and 100 mmHg. Patients were positioned in a 30° head-up position and initially sedated with benzodiazepine and an opioid. Phenytoin was given for seven days as prophylaxis for early post-traumatic seizure, and a stress ulcer prophylaxis and thromboembolic prophylaxis were given as appropriate. Nutritional support was provided with early enteral feeding.

In the intraventricular ICP monitoring group, when the ICP was higher than 20 mmHg, the CSF was drained and mannitol or a diuretic was administered to maintain the ICP below this threshold. Drainage of CSF was intermittent to remove the smallest volume of fluid necessary to control ICP. The cerebral perfusion pressure (CPP) was maintained between 60 mmHg and 70 mmHg. Refractory intracranial hypertension was defined as an ICP increase to more than 30 mmHg or a reduction in CPP to less than 60 mmHg for a period of more than 15 min, along with failure to respond to the above-mentioned maximum medical treatment. If refractory intracranial hypertension occurred, a decompressive craniotomy was performed as soon as possible. For patients in the non-ICP monitoring group, ICP and CPP were not monitored; thus, the management was solely based on clinical and radiologic findings. Mannitol (0.25–1.0 g/kg) was routinely administered every 6 or 8 h to maintain osmotic pressure at 310–320 mOsm/L.

### Data collection

Data were collected from medical records by trained research staff, and the variables included were: a) demographic data, such as age, gender, race, ethnicity, and chronic conditions (comorbidities); b) injury mechanism, GCS scores at admission, pupillary reactions; c) injury severity measured by the Injury Severity Score (ISS) and Abbreviated Injury Scores (AIS); d) length of intensive care unit (ICU) stay, total hospital stay, mechanical ventilation days; e) duration of ICP monitoring, mannitol administration, and CSF drainage; and f) device-related complications.

The CT scans obtained at admission were reviewed by two independent physicians and were classified according to the Marshall scale. The Marshall classification of TBI is based on a review of CT scans, with *diffuse injury I* indicating no visible pathology; *diffuse injury II* indicating the presence of cisterns, with a midline shift of 0 to 5 mm; *diffuse injury III* indicating pathology similar to that in diffuse injury II, but with swelling; and *diffuse injury IV* indicating pathology similar to that seen in diffuse injuries II or III, with a midline shift of more than 5 mm [19].

### Outcome assessment

The primary outcome of this study was in-hospital mortality. Secondary outcomes included the Glasgow Outcome Scale (GOS) score 6 months later, ICU and total hospital length of stays, and mechanical ventilation days. The 6-month GOS score was obtained by outpatient follow-up or telephone interview.

### Statistical analysis

All statistical analyses were performed using SPSS 20.0 (IBM, New York, NY). Unless stated otherwise, a *P* value <0.05 was considered statistically significant. Continuous variables were expressed as the mean and standard deviation. Categorical variables were described as numbers and percentages. The univariate analyses of categorical data were performed with a chi-square test. Normally distributed variables were compared using the Student t-test; whereas, non-normally distributed variables were compared using the Mann-Whitney U-test.

## Results

### Patient demographics and early characteristics

**Fig. 1:**
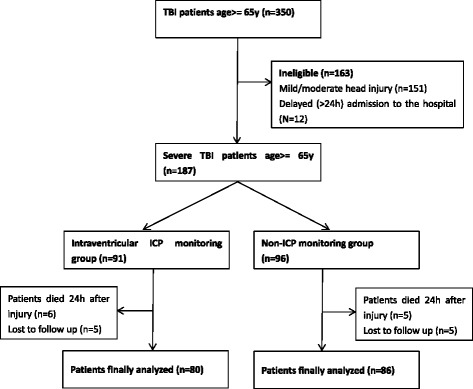
Flowchart of patient screening and study inclusion. TBI = traumatic brain injury; ICP = intracranial pressure monitoring

A total of 166 patients (aged ≥65 years) with a severe TBI, admitted to the neurosurgical department of the study hospital between January 2008 and June 2014, were enrolled in our study. Eighty patients received treatment with intraventricular ICP monitoring and 86 patients were treated without ICP monitoring (Fig. 1). The demographic and clinical characteristics of both groups are summarized in Table 1. Falls were the leading cause of head injury in both groups (53.8 and 55.8 %, respectively), followed by traffic accidents (30 and 32.6 %, respectively). The proportions of females in both groups (57.5 and 60.5 %, respectively) were higher than for males (42.5 and 39.5 %, respectively). With regard to CT findings, patients who sustained a Marshall CT classification of II were more likely to receive treatment without ICP monitoring (16.3 % vs 31.4 %, *P* < 0.05). Age, gender, comorbidities, GCS at admission, mechanism of head injury, ISS, and AIS of the head did not differ significantly between the study groups.

**Table 1:** Demographic and baseline characteristics of the study population

	ICP monitoring group	Non-ICP monitoring group	*P* value
Number of patients	80	86	
Age (years)			0.29
Median	74	76	
Interquartile range	68–78	69–82	
Gender: female, *n* (%)	46 (57.5)	52 (60.5)	0.71
Chronic conditions, *n* (%)			
Hypertension	42 (52.5)	48 (55.8)	0.79
Coronary heart disease	31 (38.8)	25 (29.1)	0.25
Pulmonary disease	12 (15.0)	11 (12.8)	0.85
Diabetes mellitus	10 (12.5)	12 (14.0)	0.95
GCS on admission, *n* (%)			0.64
6–8	56 (70.0)	64 (74.4)	
3–5	24 (30.0)	22 (25.6)	
Mechanism of head injury, *n* (%)			
Falls	43 (53.8)	48 (55.8)	0.91
Traffic accident	24 (30.0)	28 (32.6)	0.85
Others	13 (16.2)	10 (11.6)	0.53
Marshall classification on initial CT, *n* (%)			
Diffuse injury II,	13 (16.3)	27 (31.4)	0.037
Diffuse injury III,	28 (35.0)	20 (23.5)	0.14
Diffuse injury IV,	18 (22.5)	17 (19.8)	0.81
Evacuated mass lesion	9 (11.3)	8 (9.3)	0.87
Nonevacuated mass lesion	12 (14.9)	14 (16.0)	0.98
Abbreviated injury scale for head, Mean ± SD	3.78 ± 0.92	3.80 ± 0.91	0.89
Injury Severity Score (ISS), Mean ± SD	27.5 ± 9.2	28.4 ± 9.5	0.54

The Marshall classification of traumatic brain injury is based on a review of CT scans [1]

### Outcomes

Clinical outcomes are summarized in Table 2. The in-hospital mortality of the intraventricular ICP monitoring group was significantly lower than that of the non-ICP monitoring group (33.8 % vs 51.2 %, *P* < 0.05). The 6-month GOS score was also higher in the ICP monitoring group (3.0 ± 1.4 vs 2.5 ± 1.2 *P* < 0.05). However, patients with intraventricular ICP monitoring had longer ICU stays (14.3 ± 6.4 days vs 11.6 ± 5.8 days, *P* < 0.01) and longer mechanical ventilation times (6.7 ± 3.5 days vs 5.6 ± 2.4 days, *P* < 0.01) than patients without ICP monitoring. There was no significant difference in the total length of hospital stay between the two groups (28.5 ± 12.1 days vs 26.1 ± 13.5 days, *P* = 0.23).

**Table 2:** Clinical outcomes with and without ICP monitoring in older patients with severe traumatic brain injury

	ICP monitoring group	Non-ICP monitoring group	*P* value
Number of patients	80	86	
In-hospital mortality, *n* (%)	27 (33.8)	44 (51.2)	0.035
6-month GOS, Mean ± SD	3.0 ± 1.4	2.5 ± 1.2	0.014
Length of ICU stay (days), Mean ± SD	14.3 ± 6.4	11.6 ± 5.8	0.004
Length of total hospital stay (days), Mean ± SD	28.5 ± 12.1	26.1 ± 13.5	0.23
Length of mechanical ventilation (days), Mean ± SD	6.7 ± 3.5	5.6 ± 2.4	0.019

### Mannitol administration and CSF drainage

The average dose of mannitol administered in the intraventricular ICP-monitoring group was significantly lower than that used in the non-ICP monitoring group (514 ± 246 g vs 840 ± 323 g, *P* < 0.0001). In addition, the duration of mannitol use was shorter in the ICP monitoring group (6.7 ± 3.6 days vs 8.4 ± 4.3 days, *P* < 0.01). The average volume of CSF drainage was 705 ± 321 ml during a period of intraventricular ICP monitoring (7.2 ± 4.3 days) (Table 3).

**Table 3:** Mannitol administration, duration of ICP monitoring and cerebrospinal fluid drainage in two groups

	ICP monitoring group	Non-ICP monitoring group	*P*-value
Dosage of mannitol (g), Mean ± SD	514 ± 246	840 ± 323	<0.0001
Duration of mannitol treatment (days), Mean ± SD	6.7 ± 3.6	8.4 ± 4.3	0.007
Duration of ICP monitoring (days), Mean ± SD	7.2 ± 4.3	–	–
Cerebrospinal fluid drainage (ml), Mean ± SD	705 ± 321	–	–

### Device-related complications

The ICP monitoring-related complications including true problems such as catheter-induced hemorrhage and infections and device malfunctions like catheter obstruction. The incidence rates of infections and hemorrhage were 3.8 and 8.7 %, respectively. These complications were managed successfully with conservative treatment. Six of the 80 (7.5 %) ICP monitoring catheters eventually ceased draining because of obstruction. Four of the six catheters became obstructed after the 7^th^ monitored day and the other two catheters were obstructed on the 4^th^ and 5^th^ monitored days (Table 4).

**Table 4:** Device related complications after intraventricular ICP monitoring

	ICP monitoring group	Non-ICP monitoring group	*P*-value
Ceased draining because of catheter obstruction, *n* (%)	6 (7.5)	–	–
Infections, *n* (%)	3 (3.8)	–	–
Hemorrhage, *n* (%)	7 (8.7)	–	–

## Discussion

The aim of this study was to determine the effect of intraventricular ICP monitoring on outcome of elder patients with a severe TBI. The results indicated that elder severe TBI patients managed with intraventricular ICP monitoring had lower in-hospital mortality and improved 6-month functional outcome compared with patients without ICP monitoring. However, the length of ICU stay and mechanical ventilation duration were longer in ICP monitored patients. Although ICP monitoring has long been considered the standard of care for severe TBI patients and is recommended by several guidelines [5–7], there are still debates over the effect of ICP monitoring on the outcome of severe TBI patients. Two retrospective studies and a prospective study concluded that ICP monitoring was associated with decreased in-hospital or two-week mortality [8–10]. Cremer et al. conducted a large prospective cohort study in two medical centers, patients in center A received supportive care treatment while patients in center B were treated under the ICP/CPP target protocol (maintaining ICP <20 mmHg and CPP >70 mmHg). They found no evidence for improved outcome in patients who received ICP monitoring despite a higher treatment intensity [12]. Mauritz et al. performed a prospective multi-center study in Australia, and found the lowest mortality rate in the subgroup with the highest rate (91.1 %) of ICP monitoring. However, the differences were not significant. Worsening outcome in severe TBI patients with ICP monitoring was also reported in a retrospective study [13]. Nevertheless, previous observational studies have generally suffered from methodological weaknesses, including: selection bias, difference in treatment protocols, baseline differences between groups in injury severity, and comorbidities that may cofound the outcomes. Due to ethical considerations, Chesnut et al. conducted the only randomized control trial about ICP monitoring in severe TBI patients, to date. The trial was conducted in Latin America, where ICP monitoring was not the standard of care in most hospitals. No difference was found in the primary outcome, which was a composite of 21 measures including survival time and 6-month functional and neuropsychological status, between patients with or without ICP monitoring [11]. However, some issues about this trial warrant discussion. First, this trial was conducted in South America, where the medical system and availability of resources, including pre-hospital resuscitation and care after hospital discharge, were less developed than those in higher income countries [20, 21]. Second, ICP was monitored by intraparenchymal monitors and CSF drainage was rarely used in this trial [20]. Finally, the elderly TBI patients, who are prominent in developed countries, were not present in this trial [11].

In our study, patients treated with intraventricular ICP monitoring had lower in-hospital mortality (33.8 % vs 51.2 %, *P* < 0.05) and better 6-month functional outcome (3.0 ± 1.4 vs 2.5 ± 1.2, *P* < 0.05) than patients without ICP monitoring. Our findings, in conjunction with multiple studies reporting on the positive effects of ICP monitoring, add to the beneficial evidence of ICP monitoring in the management of severe TBI, especially in the elderly population. Poorer outcomes after brain injury were often observed among elder adults (aged ≥65 years) compared with younger patients. Evidence suggested that elderly patients received less aggressive treatment, including ICP monitoring, than younger adults [22, 23]. Nevertheless, previous studies have demonstrated that it would be beneficial to increase the treatment intensity for this group of patients, and elder patients may be more responsive to treatment for lowering intracranial hypertension [24, 25]. With ICP monitoring, information gained allows for more informed decisions regarding the management of elderly TBI patients.

There are currently two major approaches for continuous ICP monitoring, intraparenchymal and intraventricular, each with its own merits and drawbacks [16, 26]. The benefits of ICP monitoring include maintaining an adequate CPP, providing an early indication for surgical management, and guiding the administration of therapy for lowering ICP [16]. The evidence for the association between types of ICP monitoring and outcome in TBI patients are variable [16, 17]. Intraventricular ICP monitoring was chosen in this study since it is considered the most reliable and accurate method of measuring ICP. Moreover, intraventricular catheters can also provide an effective means of lowering ICP by drainage of CSF. By draining CSF with an average volume of 705 ± 321 ml in this study, the ICP monitoring group received a significantly lower dose of mannitol (514 ± 246 g vs 840 ± 323 g, *P* < 0.0001) and shorter duration of mannitol administration (6.7 ± 3.6 days vs 8.4 ± 4.3 days, *P* < 0.01) than the control group. It has been established that mannitol may cause hypovolemia and result in episodes of hypotension, which has been associated with increased mortality in brain injury patients [27, 28]. Due to its nephrotoxicity, mannitol can lead to acute kidney injury (AKI) [29]. A study conducted by Zeng J et al. suggested that intraventricular ICP monitoring reduced the incidence of AKI in TBI patients by reducing the use of mannitol [30]. Often presented with multiple pre-existing comorbidities, elderly TBI patients were at higher risk of developing secondary complications, such as cardiac arrest and acute renal failure, than younger adults [3]. By reducing the use of mannitol, the beneficial effect of CSF drainage via intraventricular catheters is amplified in the group of older TBI patients. This may be another reason for the better outcome observed in the ICP monitoring group.

With regard to the in-hospital mortality, our results (overall mortality 42.8 %) were lower than those reported in previous studies. The mortality of severe TBI patients aged 65 years or older in other studies ranged from 72 to 83.3 % [31–33]. The lower mortality in our cohort than other studies could be attributed to the exclusion of the “worst cases” (patients died within 24 h of brain injury or admitted with a diagnosis of brain death) in this study.

In this study, the length of ICU stay (14.3 ± 6.4 days vs 11.6 ± 5.8 days, *P* < 0.01) and duration of mechanical ventilation (6.7 ± 3.5 days vs 5.6 ± 2.4 days, *P* < 0.05) were significantly longer in the ICP monitoring group. However, conflicting results about this topic have been reported previously [9, 14, 21, 34]. The differing results about the length of ICU stay can potentially be attributed to the types of ICP monitoring used. Intraventricular ICP monitoring was previously associated with longer ICU lengths of stay than intraparenchymal ICP monitoring [16]. The ICP monitoring was performed under general anesthesia; thus, the mechanical ventilation days were significantly longer in the ICP monitoring group. Nevertheless, the overall hospital stay (28.5 ± 12.1 days vs 26.1 ± 13.5 days, *P* = 0.23), a parameter more reflective of injury burden, did not differ between groups in this study. This can be explained by the similar injury severities between groups.

The complications associated with intraventricular ICP monitoring were also analyzed in this study. Six of the 80 (7.5 %) catheters were eventually obstructed and ceased draining, and this is lower than the rates reported previously [16]. In our study, most of the catheters (4 of 6) became obstructed after the 7^th^ monitored day without significantly affecting the drainage of CSF. Device-related infections were seldom observed in this study, with an occurrence rate of 3.8 %. This was likely due to the routine prophylactic antibiotic treatment in all patients who underwent ICP monitoring in our department. Hemorrhage associated with catheter insertion was identified in 8.7 % of patients, but all the cases were managed successfully with conservative treatment.

There are several potential limitations of this study. First, this was an observational study conducted in a single institution, and there are inherent limitations of this type of study. Second, the study was not randomized, which may reduce its statistical power. Third, the decision-making process for intraventricular ICP monitoring was not entirely standardized. Some patients in the non-ICP monitoring group met the criteria, but did not undergo ICP monitoring due to other factors. Thus, further well-designed, multicenter studies are needed to confirm the study results.

## Conclusion

In our study, older severe TBI patients who underwent intraventricular ICP monitoring had lower in-hospital mortality and improved 6-month outcomes compared with patients without ICP monitoring. These favorable findings may be attributed to intraventricular ICP monitoring. However, given the limitations of this study, further well-designed randomized control trials are needed to confirm the beneficial effects of intraventricular ICP monitoring on the outcomes of older severe TBI patients.

## Abbreviations

AIS, Abbreviated Injury Scores; AKI, acute kidney injury; CPP, cerebral perfusion pressure; CSF, cerebrospinal fluid; GCS, Glasgow Coma Scale; GOS, Glasgow Outcome Scale; ICP, intracranial pressure; ICU, intensive care unit; ISS, Injury Severity Score; TBI, traumatic brain injury

## References

[1] Hyder AA, Wunderlich CA, Puvanachandra P, Gururaj G, Kobusingye OC (2007). The impact of traumatic brain injuries: a global perspective. NeuroRehabilitation.

[2] Susman M, DiRusso SM, Sullivan T, Risucci D, Nealon P, Cuff S (2002). Traumatic brain injury in the elderly: increased mortality and worse functional. J Trauma.

[3] Dams-O’Connor K, Cuthbert JP, Whyte J, Corrigan JD, Faul M, Harrison-Felix C (2013). Traumatic brain injury among older adults at level I and II trauma centers. J Neurotrauma.

[4] Livingston DH, Lavery RF, Mosenthal AC, Knudson MM, Lee S, Morabito D (2005). Recovery at one year following isolated traumatic brain injury: a Western Trauma Association prospective multicenter trial. J Trauma.

[5] Bratton SL, Chestnut RM, Ghajar J, McConnell Hammond FF, Harris OA, Hartl R (2007). Guidelines for the management of severe traumatic brain injury. III Prophylactic. J Neurotrauma.

[6] Maas AI, Dearden M, Teasdale GM, Braakman R, Cohadon F, Iannotti F (1997). EBIC-guidelines for management of severe head injury in adults. European Brain. Acta Neurochir.

[7] Procaccio F, Stocchetti N, Citerio G, Berardino M, Beretta L, Della Corte F (2000). Guidelines for the treatment of adults with severe head trauma (part I). Initial. J Neurosurg Sci.

[8] Farahvar A, Gerber LM, Chiu YL, Carney N, Hartl R, Ghajar J (2012). Increased mortality in patients with severe traumatic brain injury treated without intracranial pressure monitoring. J Neurosurg.

[9] Lane PL, Skoretz TG, Doig G, Girotti MJ (2000). Intracranial pressure monitoring and outcomes after traumatic brain injury. Can J Surg.

[10] Bulger EM, Nathens AB, Rivara FP, Moore M, MacKenzie EJ, Jurkovich GJ (2002). Management of severe head injury: institutional variations in care and effect on outcome. Crit Care Med.

[11] Chesnut RM, Temkin N, Carney N, Dikmen S, Rondina C, Videtta W (2012). A trial of intracranial-pressure monitoring in traumatic brain injury. N Engl J Med.

[12] Cremer OL, van Dijk GW, van Wensen E, Brekelmans GJ, Moons KG, Leenen LP (2005). Effect of intracranial pressure monitoring and targeted intensive care on. Crit Care Med.

[13] Shafi S, Diaz-Arrastia R, Madden C, Gentilello L (2008). Intracranial pressure monitoring in brain-injured patients is associated with worsening of survival. J Trauma.

[14] Tang A, Pandit V, Fennell V, Jones T, Joseph B, O’Keeffe T (2015). Intracranial pressure monitor in patients with traumatic brain injury. J Surg Res.

[15] Yuan Q, Wu X, Yu J, Sun Y, Li Z, Du Z (2015). Effects and clinical characteristics of intracranial pressure monitoring-targeted management for subsets of traumatic brain injury: an observational multicenter study. Crit Care Med.

[16] Kasotakis G, Michailidou M, Bramos A, Chang Y, Velmahos G, Alam H (2012). Intraparenchymal vs extracranial ventricular drain intracranial pressure monitors. J Am Coll Surg.

[17] Liu H, Wang W, Cheng F, Yuan Q, Yang J, Hu J (2015). External ventricular drains versus intraparenchymal intracranial pressure monitors in traumatic brain injury: a prospective observational study. World Neurosurg.

[18] Brain Trauma F, Bratton SL, American Association of Neurological S, Congress of Neurological S, Joint Section on N, Critical Care AC (2007). Guidelines for the management of severe traumatic brain injury. VI. Indications for intracranial pressure monitoring. J Neurotrauma.

[19] Marshall LF, Marshall SB, Klauber MR, Van Berkum Clark M, Eisenberg HM, Jane JA (1991). A new classification of head injury based on computerized tomography. J Neurosurg.

[20] Sarrafzadeh AS, Smoll NR, Unterberg AW (2014). Lessons from the intracranial pressure-monitoring trial in patients with traumatic brain injury. World Neurosurg.

[21] Melhem S, Shutter L, Kaynar A (2014). A trial of intracranial pressure monitoring in traumatic brain injury. Crit Care.

[22] Munro PT, Smith RD, Parke TR (2002). Effect of patients’ age on management of acute intracranial haematoma. BMJ.

[23] Mauritz W, Janciak I, Wilbacher I, Rusnak M (2007). Severe traumatic brain injury in Austria IV: intensive care management. Wien Klin Wochenschr.

[24] Farahvar A, Gerber LM, Chiu YL, Härtl R, Froelich M, Carney N (2011). Response to intracranial hypertension treatment as a predictor of death in. J Neurosurg.

[25] Whitmore RG, Thawani JP, Grady MS, Levine JM, Sanborn MR, Stein SC (2012). Is aggressive treatment of traumatic brain injury cost-effective?. J Neurosurg.

[26] Bratton SL, Chestnut RM, Ghajar J, McConnell Hammond FF, Harris OA, Hartl R (2007). VII. Intracranial pressure monitoring technology. J Neurotrauma.

[27] Manley G, Knudson MM, Morabito D, Damron S, Erickson V, Pitts L (2001). Hypotension, hypoxia, and head injury: frequency, duration, and consequences. Arch Surg.

[28] Jeremitsky E, Omert L, Dunham CM, Protetch J, Rodriguez A (2003). Harbingers of poor outcome the day after severe brain injury: hypothermia. J Trauma.

[29] Pérez-Pérez AJ, Pazos B, Sobrado J, Gonzalez L, Gándara A (2002). Acute renal failure following massive mannitol infusion. Am J Nephrol.

[30] Zeng J, Tong W, Zheng P (2013). Decreased risk of acute kidney injury with intracranial pressure monitoring in patients with moderate or severe brain injury. J Neurosurg.

[31] Hukkelhoven CW, Steyerberg EW, Rampen AJ, Farace E, Habbema JD, Marshall LF (2003). Patient age and outcome following severe traumatic brain injury: an analysis of. J Neurosurg.

[32] McIntyre A, Mehta S, Aubut J, Dijkers M, Teasell RW (2013). Mortality among older adults after a traumatic brain injury: a meta-analysis. Brain Inj.

[33] Utomo WK, Gabbe BJ, Simpson PM, Cameron PA (2009). Predictors of in-hospital mortality and 6-month functional outcomes in older. Injury.

[34] Alkhoury F, Kyriakides TC (2014). Intracranial pressure monitoring in children with severe traumatic brain injury: National Trauma Data Bank-Based Review of Outcomes. JAMA Surg.

